# CD11c+ dendritic cells PlexinD1 deficiency exacerbates airway hyperresponsiveness, IgE and mucus production in a mouse model of allergic asthma

**DOI:** 10.1371/journal.pone.0309868

**Published:** 2024-08-30

**Authors:** Lianyu Shan, Mojdeh Matloubi, Ifeoma Okwor, Sam Kung, Mohamed Sadek Almiski, Sujata Basu, Andrew Halayko, Latifa Koussih, Abdelilah S. Gounni

**Affiliations:** 1 Department of Immunology, Rady Faculty of Health Sciences, Max Rady College of Medicine, University of Manitoba, Winnipeg, Manitoba, Canada; 2 Department of Anatomy, Rady Faculty of Health Sciences, Max Rady College of Medicine, University of Manitoba, Winnipeg, Manitoba, Canada; 3 Depertment of Physiology and Pathophysiology, Rady Faculty of Health Sciences, Max Rady College of Medicine, University of Manitoba, Winnipeg, Manitoba, Canada; 4 Department of Experimental Biology, Université de Saint-Boniface, Winnipeg, Manitoba; University of Maryland School of Medicine, UNITED STATES OF AMERICA

## Abstract

Dendritic cells (DCs) are pivotal in regulating allergic asthma. Our research has shown that the absence of Sema3E worsens asthma symptoms in acute and chronic asthma models. However, the specific role of PlexinD1 in these processes, particularly in DCs, remains unclear. This study investigates the role of PlexinD1 in CD11c+ DCs using a house dust mite (HDM) model of asthma. We generated CD11c+ DC-specific PlexinD1 knockout (*CD11c*^*PLXND1 KO*^) mice and subjected them, alongside wild-type controls (*PLXND1*^fl/fl^), to an HDM allergen protocol. Airway hyperresponsiveness (AHR) was measured using FlexiVent, and immune cell populations were analyzed via flow cytometry. Cytokine levels and immunoglobulin concentrations were assessed using mesoscale and ELISA, while collagen deposition and mucus production were examined through Sirius-red and periodic acid Schiff (PAS) staining respectively. Our results indicate that *CD11c*^*PLXND1 KO*^ mice exhibit significantly exacerbated AHR, characterized by increased airway resistance and tissue elastance. Enhanced mucus production and collagen gene expression were observed in these mice compared to wild-type counterparts. Flow cytometry revealed higher CD11c+ MHCII^high^ CD11b+ cell recruitment into the lungs, and elevated total and HDM-specific serum IgE levels in *CD11c*^*PLXND1 KO*^ mice. Mechanistically, co-cultures of B cells with DCs from *CD11c*^*PLXND1 KO*^ mice showed significantly increased IgE production compared to wild-type mice.These findings highlight the critical regulatory role of the plexinD1 signaling pathway in CD11c+ DCs in modulating asthma features.

## Introduction

Asthma is a significant public health problem, affecting more than 358 million individuals worldwide [1]. Allergic asthma is a chronic inflammatory disorder involving the airways, with a predominant Th2/Th17 immune response and an increase of innate and adaptive immune cells. Inflammatory cells release various mediators, such as cytokines, chemokines, histamine, immunoglobulins (Igs), growth factors, and lipid mediators, which contribute to the development of airway hyperreactivity, excessive mucus production, collagen deposition, hypertrophy and hyperplasia of airway smooth muscle (ASM), and subsequent changes in the airway architecture or remodelling [2]. Despite advancements in drug therapies, approximately 5–10% of asthma patients remain unresponsive to these medications [3], highlighting the crucial need for novel therapeutic approaches and a deeper understanding of the underlying factors regulating the pathophysiology of asthma.

Initially identified as axon guidance cues in neural development [4], semaphorins are now known to be widely expressed across various organs and tissues, where they participate in diverse signaling pathways [5, 6]. In pulmonary diseases, semaphorins are crucial for cell-cell contact, migration, proliferation, differentiation, and immune regulation [7, 8]. Notably, Semaphorin3E (Sema3E) has emerged as a significant regulatory molecule in asthma, influencing both the immune response and airway remodeling [9].

Sema3E binds with high affinity to its canonical receptor, PlexinD1 [10, 11], a key regulator of axon guidance, vascular patterning, and thymocyte migration [7, 11]. Loss of PlexinD1 function has been linked to various autoimmune diseases and cancers [12], highlighting its importance in immune regulation and cellular signaling.

Previous research from our group has demonstrated the pivotal roles of Sema3E and its receptor PlexinD1 in regulating airway inflammation, remodeling, and hyperresponsiveness in asthma [9, 13–20]. Specifically, global deletion of the *Sema3E* gene in mice resulted in significant lung granulocyte recruitment, accompanied by a pronounced Th2/Th17 immune response [14, 16]. This was characterized by increased airway hyperresponsiveness (AHR), excessive mucus production, and collagen deposition, leading to sub-epithelial fibrosis. In contrast, intranasal administration of recombinant Sema3E mitigated these pathological changes, highlighting the essential homeostatic function of the Sema3E-PlexinD1 signaling axis in allergic asthma [9].

Dendritic cells (DCs) are crucial links between innate and adaptive immunity, acting as primary antigen-presenting cells that initiate and modulate immune responses [21]. Their pivotal role in immune regulation is particularly significant in inflammatory conditions, including asthma, where DC function directly impacts immune response initiation, progression, and resolution. Consequently, understanding the mechanisms that regulate DC functions is critical for developing new therapeutic strategies for inflammatory diseases [21].

Sema3E plays a significant role in modulating DC behavior in allergic asthma [17, 18]. Using a mouse model of HDM-induced allergic asthma, we revealed that *Sema3E*-deficient (KO) mice exhibited an increased population of CD11c+ CD11b+ CD103- DCs, which consequently led to Th2/Th17 immune activation compared to wild-type mice [18]. Furthermore, HDM sensitization in Sema3E KO mice resulted in elevated expression of programmed death-ligand 2 (PDL2), interferon regulatory factor 4 (IRF-4) CC chemokine receptor-7 (CCR7) and enhanced allergen uptake capacity in pulmonary CD11b+ DCs, compared to their wild-type counterparts [17]. *In vitro* experiments using Sema3E KO CD11c+ bone marrow-derived dendritic cells (BMDCs) demonstrated increased baseline migration associated with an enhanced Rac1 GTPase activity and actin polymerization in response to CCL21, in comparison to DCs isolated from wild-type mice. These findings suggest that the absence of Sema3E may augment the ability of DCs to uptake HDM allergens and migrate to the lymph nodes, subsequently leading to enhanced activation of T and B cells and further exacerbation of allergic asthma-associated inflammation [17].

Moreover, upon the adoptive transfer of CD11b+ DCs from Sema3E-KO mice to wild-type mice, we observed the recapitulation of the abovementioned responses in the wild-type recipient mice [18]. In light of these findings, we conclude that Sema3E is a guidance cue for recruiting lung DCs and modulates T and B cell responses in allergic asthma. Nevertheless, the role of plexinD1 in these events needs to be clarified. Therefore, we investigated the role of plexinD1 deficient CD11c+ DC in a murine model of allergic asthma. In this study, we found that deficiency of *PLXND1* in CD11c+ DC exacerbates airway hyperresponsiveness (AHR), enhances mucus production, and upregulates collagen gene expression. Moreover, the absence of *PLXND1* in CD11c+ DC leads to the accumulation of conventional DC in the lungs, along with increased IgE levels and overall inflammation. These findings indicate that plexinD1 in CD11c+ DC plays a crucial regulatory role in allergic asthma features.

## Method and materials

### Animals

*PLXND1*^*fl/fl*^ mice (B6;129-*PLXND1*^*tm1*.*1Tmj/J*^) [22] were kindly provided by Dr. T.M. Jessell (Columbia University/Howard Hughes Medical Institute, New York, NY) and were crossed with *B6*.*Cg-Tg*(*Itgax-cre)*^*1-1Reiz/J*^ mice (The Jackson Laboratory, stock number 008068). The latter mice express Cre recombinase under the control of the mouse integrin alpha X (CD11c) promoter, resulting in the generation of *CD11c(Itgax-cre)*:*PLXND1*^*fl/fl*^ mouse.

To assess immune cell parameters, mice were anesthetized with an overdose of isoflurane. Anesthesia was induced by placing the mice in an induction chamber with the gas flowmeter set at 500–1000 ml/min and the vaporizer at 4%. Anesthesia was confirmed by the absence of pedal reflex. For the procedure, a nosecone maintained anesthesia with the flowmeter at 100–200 ml/min and the vaporizer at 2–3%, ensuring the mice felt no pain. Mice were euthanized by cardiac perfusion or cervical dislocation.

All mice were housed in the pathogen-free room at the Central Animal Care facility at the University of Manitoba. All procedures followed the guidelines provided by the Canadian Council for Animal Care and were approved by the University of Manitoba Animal Care and Use Committee (protocol number 19–035).

### HDM-induced airway inflammation model

Six- to eight-week-old female *PLXND1*^*fl/fl*^ (wild-type) and *CD11c*^*PLXND1 KO*^ mice were administered 25 mg of HDM extract (lot 259585; Greer Laboratories, Lenoir, NC) intranasally in 35μl of sterile saline, five days per week for two consecutive weeks, under gaseous anesthesia [18]. Wild-type and *CD11c*^*PLXND1 KO*^ control mice were challenged with 35μl of sterile saline. Mice were sacrificed 48 hours after the final HDM challenge to assess airway inflammation and other outcomes.

### Methacholine challenge test

Airway hyperresponsiveness (AHR) parameters, including airway resistance (Rn), tissue resistance (G), and tissue elastance (H), were evaluated using the FlexiVent animal ventilator (Scireq, Montreal, QC, Canada). Mice were anesthetized with intraperitoneal pentobarbital sodium (54.7 mg/mL). Each mouse received a 90 mg/kg dose, calculated based on their body weight (e.g., 33 μL for a 20 g mouse). Anesthesia was confirmed by the lack of a pedal withdrawal reflex. If necessary, a 45 mg/kg maintenance dose was administered to ensure a proper surgical plane of anesthesia. Once anesthetized, a tracheotomy was performed.

Mice that received HDM or saline underwent thoracotomy, followed by intratracheal administration of an increasing gradient of methacholine dose (Saline, 3, 6, 12, 25, and 50 mg/ml) at 5-minute intervals. Lung functions were investigated as previously described [14].

### Bronchoalveolar lavage fluid collection and differential cell count

Bronchoalveolar lavage fluid (BALF) was collected by instilling 1 mL of sterile PBS containing 0.05 mM EDTA into the airways twice. The fluid was centrifuged, and the supernatant was stored at −80°C for later analysis. Total BALF cells were counted using trypan blue exclusion and a hemocytometer. For differential cell counts, cells were prepared on cytospin slides, fixed, and stained with Wright-Giemsa. Two independent observers, blinded to the experimental groups, performed differential counts on 200 cells per sample.

### Immunophenotyping of BALF, spleen, blood, lymph node, and lung immune cells

Lungs, lymph nodes, blood, and spleen cells were utilized for immunophenotyping using FACS under the steady state condition. Tissues from *PLXND1*^*fl/fl*^ and *CD11c*^*PLXND1 KO*^ mice were collected, and single-cell suspensions were prepared [18]. Following washing and blocking with Fc-blocker, cells were stained with a mixture (0.5μl of antibodies/20μl of flow buffer per tube) containing the following anti-mouse antibodies using two antibody panels. The first panel consisted of fixable viability dye eFluor 780 (eBioscience), Siglec F-PE (clone E50-2440; BD Biosciences), CD11b-PE/Cy7 (clone M1/70), CD11c-PerCP/Cy5.5 (clone N418), Ly6G-allophycocyanin (clone 1A8), F4/80-FITC (clone BM8; all four from BioLegend). The second panel included fixable viability dye eFluor 780 (eBioscience), NK1.1-PE/Cy7 (clone PK136; eBioscience), CD3-PE (clone 145-2C11; eBioscience), CD4-allophycocyanin (clone GK1.5; Biolegend), and B220-FITC (clone RA3-6B2; BD Biosciences).

Moreover, inflammatory cells in the BALF were characterized using anti-mouse antibodies, including Siglec-F PE (clone E50-2440; BD Biosciences), CD11c Percp/Cy5.5 (clone N418), Ly6G-allophycocyanin (clone 1A8), CD11b PE/Cy7 (clone M1/70), F4/80 FITC (clone BM8; all four from BioLegend), and fixable viability dye APC-Cy7 (eBioscience). Subsequently, the samples were acquired using a BD FACSCanto II flow cytometer and analyzed using FlowJo software.

### Analyzing lung DC subsets and the expression of costimulatory molecules

Lungs were collected from *CD11c*^*PLXND1 KO*^ and *PLXND1*^*fl/fl*^ mice challenged with either saline or HDM. The whole lung was minced and enzymatically digested in RPMI 1640 medium containing 1 mg/ml collagenase IV (Worthington Biochemical, Lakewood, NJ) at 37°C for 30 min. After red blood cell lysis with ACK (ammonium-chloride-potassium) buffer, the cells were counted and stained with anti-mouse antibodies (0.5μl of antibodies/20μl of flow buffer per tube) after Fc blocking. The antibody mixture included fixable viability dye eFluor 780 (eBioscience), CD45-eFluor 450 (clone 30F11), F4/80-FITC (clone BM8; eBioscience), anti-mouse CD11c-allophycocyanin (clone 418; eBioscience), MHC class II (I-A/I-E) eFluor 450 (clone M5/114.15.2; eBioscience), CD11b PE-Cy7 (clone M1/70; BioLegend), CD103 PerCP-Cy5.5 (clone 2E7; BioLegend), CD40-BV605 (clone 5C3; BioLegend), CD80-APC (clone 16-10A1; BioLegend), and CD86- APC-Cy7 (clone GL-1; BioLegend). Subsequently, the samples were acquired as described above.

### DC differentiation and isolation from bone marrow

Bone marrow was collected from naive *CD11c*^*PLXND1 KO*^ and *PLXND1*^*fl/fl*^ mice. Red blood cells were lysed using ammonium chloride solution (ACK lysis buffer solution). Bone marrow cells were cultured in RPMI 1640 culture medium (Sigma, St Louis, MO, USA) supplemented with 10% fetal calf serum (FCS), 1% MEM non-essential amino acids solution (GIBCO, Berlin, Germany), 1% penicillin-streptomycin (GIBCO), 1% HEPES buffer solution (GIBCO), 1% sodium pyruvate (GIBCO), 50 lM 2-mercaptoethanol (GIBCO) and 20 ng/ml recombinant mouse granulocyte-macrophage colony-stimulating factor (GM-CSF) (PEPRO TECH EC LTD, London, UK). The cells were incubated at 37°C with 5% CO2 for six days, with medium changed every two days.

Mature bone marrow-derived dendritic cells (mBMDCs) were obtained by overnight stimulation with 1μg/ml lipopolysaccharide (LPS) (Sigma). Conventional DCs were then sorted using fixable viability dye APC-Cy7 (eBioscience), anti-mouse CD11c-allophycocyanin (clone 418; eBioscience), MHC class II (I-A/I-E) eFluor 450 (clone M5/114.15.2; eBioscience) on a FACS flow cytometer. The purity of the sorted cells was confirmed to be greater than 92%, as checked by flow cytometry using a FACS Calibur (BD Biosciences, San Jose, CA) [23].

### B cell isolation and co-culture with BMDC

B cell co-culture with DCs was performed as previously described [23, 24]. In brief, a spleen was harvested from a wild-type mouse, and single-cell suspension was prepared by lysing red blood cells using ammonium chloride solution (ACK lysis buffer solution). B cells were then isolated using the EasySep™ Mouse B Cell Isolation Kit (Stemcell Technologies). The purity of the isolated B cells was consistently above 95%, as determined by flow cytometry using a FACS Calibur instrument.

For the co-culture experiment, 50 μl of B cells at a concentration of 2 x 10^6^ cells/ml and 50 μl of BMDCs at 2 x 10^6^ cells/ml (1:1 ratio) were co-cultured for four days in the presence of 0.5ug/ml purified NA/LE hamster anti-mouse CD40 (clone HM40-3 BD Pharmingen), 10 μg/ml affinity pure F(ab)2 fragment goat anti-mouse IgM (clone 115-006-020; Jackson ImmunoResearch), and 25 ng/ml recombinant mouse IL-4 (clone 404-ML-025/CF; R&D System) in final volume of 200 μl of RPMI 1640 medium supplemented with 10% FBS, 2 mM L-glutamine, 100 U/ml penicillin,100 U/ml streptomycin, and 50 mM 2-ME, in 96-well plates. After four days, the supernatant was collected, and IgE production was measured using ELISA [23].

### Cytokine measurement

Mesoscale ELISA was used to assess IL-4, IL-5, IL-13, IL-17A, IFN-γ, and CCL-2/MCP-1 levels in BALF supernatants according to the manufacturer’s instructions. ELISA data was analyzed using SoftMax Pro software (Molecular Devices). All cytokine ELISA kits were purchased from BioLegend (San Diego, CA), except for IL-13 (eBioscience).

### Intracellular cytokine detection

The intracellular staining of cytokines was conducted as previously described [14]. Briefly, mediastinal lymph node cells were cultured for 4 hours at 37°C with 5% CO2 and stimulated with PMA, ionomycin, and the protein transport inhibitor brefeldin A (Invitrogen).

Cells were then collected, and extracellular staining was performed using anti-mouse CD3 PE/Cy7 (clone 145-2C11; eBioscience) and CD4-allophycocyanin (clone G1.5; eBioscience). Subsequently, intracellular staining was performed using specific anti-mouse antibodies, including IFN-γ PerCP-Cy5.5 (clone XMG1.2; eBioscience), IL-4 PE (clone 11B11; eBioscience), and IL-17A (clone TC11-18H10.1; BioLegend). The samples were acquired using the FACSCanto II flow cytometer and analyzed with FlowJo software.

### Measurement of immunoglobulins in serum

Cardiac blood was collected from *CD11c*^*PLXND1 KO*^ and *PLXND1*^*fl/fl*^ mice that received either saline or HDM. Following centrifugation, the serum samples were obtained to measure the total and HDM-specific IgE and IgG1 using ELISA, according to the manufacturer’s instructions [14, 25]. ELISA antibodies for measuring total and HDM-specific Igs in serum were purchased from Southern Biotech (Birmingham, AL). ELISA data were analyzed using SoftMax Pro software (Molecular Devices).

### Lung histology

The left lobe of the lung was dissected, fixed in 10% formalin overnight, and embedded in paraffin. Lung tissue sections were stained with hematoxylin and eosin (H&E) to assess inflammation, periodic acid-Schiff (PAS) to evaluate mucus production, and Sirius red to visualize collagen deposition. The severity of airway inflammation, mucus production, and collagen deposition was assessed in *CD11c*^*PLXND1 KO*^ and wild-type mice after saline or HDM administration [14]. A blinded pathologist performed pathological scoring.

To evaluate inflammation around the airways, we used a scoring system as follows: 0 points for no inflammation, 1 point for a few inflammatory cells around the airway, 2 points for a single ring of inflammatory cells, 3 points for a ring of inflammatory cells two to four cells deep, and 4 points for a ring more than four cells deep. For mucus scoring, periodic acid-Schiff (PAS)-stained sections were graded on a scale from 0 to 4 based on the percentage of the airway covered by positively stained cells: 0 points for 0% coverage, 1 point for 1–25% coverage, 2 points for 26–50% coverage, 3 points for 51–75% coverage, and 4 points for more than 75% coverage. Collagen scoring assessed fibrosis with the following grades: 0 for normal lung, 1 for minimal fibrosis with slight thickening of alveolar or bronchiolar walls, 2–3 for moderate thickening without significant damage to lung architecture, 4–5 for increased fibrosis with apparent structural damage and formation of fibrous bands or small masses, 6–7 for severe structural distortion and large fibrous areas (including "honeycomb lung"), and 8 for total fibrous obliteration of the field [26].

### Real-time PCR

Total RNA was isolated from the middle lobe of the lung using TRIzol (Ambion). MultiScribe reverse transcriptase was performed for 1μg of RNA to synthesize cDNA according to the manufacturer’s instructions (Applied Biosystems, Foster City, CA). The expression of the collagen (*COL3*) and mucin (*MUC5AC*) genes was analyzed by quantitative real-time PCR (qRT-PCR) (Table 1). Eukaryotic elongation factor 2 (EEF2) was used as a housekeeping gene. qRT-PCR was done in a 96-well optical plate with an initial one-cycle denaturation step for 10 min at 95°C, 40 cycles of PCR (95^°C^ for 15s, 60^°C^ for 30s, and 72^°C^ for 30s), one cycle of melting, and one cooling cycle (Bio-Rad CFX96 real-time PCR system). Product specificity was assessed by performing a melting curve analysis and examining the quality of amplification curves. The amplification of target genes was calculated by normalizing by the amplification of EEF2 (Δ^Ct^) and then normalizing by control groups (ΔΔ^Ct^) [27].

**Table 1:** The forward and reverse primers are used to investigate gene expression.

Gene	Forward primer	Reverse primer
*COL3*	GCAGGACCCAGAGGAGTAG	TTCCATCATTGCCTGGTC
*MUC5AC*	GCATGTTGGTACCCCACTCA	GTTGCAGAGACCAGGGAAGT

### Statistical analyses

GraphPad Prism 9.0 software was used for statistical analysis. Depending on the number of groups and treatments, data were analyzed by a one-way ANOVA or two-way ANOVA, followed by a Tukey test. Differences were statistically significant at *p < 0.05, **p < 0.01, and ***p < 0.001.

## Results

### *PLXND1* ablation in CD11c+ cells does not alter the composition of immune cells under steady-state conditions

We conducted FACS-based immunophenotyping of various immune cell populations, including neutrophils, eosinophils, alveolar macrophages (AM), interstitial macrophages (IM), NK cells, B cells, and T cells, in the lungs, spleen, mediastinal lymph nodes, and blood of *CD11c*^*PLXND1 KO*^ and *PLXND1*^*fl/fl*^ mice (wild type).

**Fig 1:**
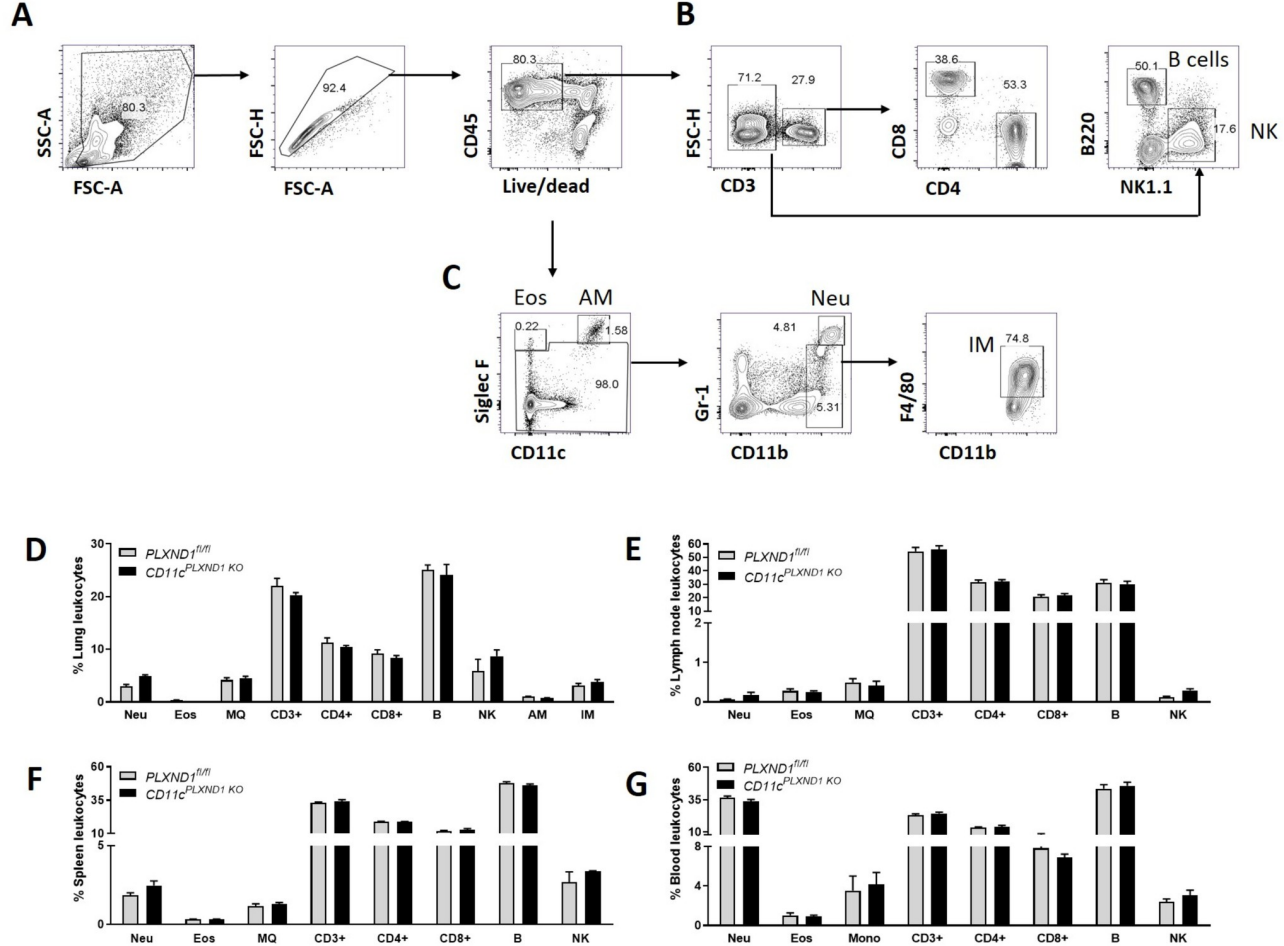
Immunophenotyping of inflammatory cells in *CD11c*^*PLXND1 KO*^ and *PLXND1*^*fl/fl*^ mice. Lungs, spleen, blood, and lymph nodes were harvested from *CD11c*^*PLXND1 KO*^ and wild-type mice. Following enzymatic digestion, single-cell suspensions were analyzed using FACS with specific antibodies to characterize various inflammatory cell populations at the steady state. **(A)** The general gating strategy excluded debris and doublets, focusing on viable leukocytes **(B)** T and B cells were identified by surface expression of CD3 and B220, followed by further identification of CD4+ and CD8+ cells within the CD3-expressing population. Pulmonary NK cells were identified as NK1.1+ cells. **(C)** Eosinophils were Siglec-F+/CD11c-, alveolar macrophages were Siglec-F+/CD11c+, neutrophils were Ly6G+ (1A8)/CD11b+, interstitial macrophages were F4/80+/CD11b+. The numbers of each cell type in **(D)** lung, **(E)** mediastinal lymph node, **(F)** spleen, and **(G)** blood were compared between *CD11c*^*PLXND1 KO*^ and WT mice under the steady-state condition. Data represent the mean (pre-gated on CD45+) with SEM. Representative of four to five mice per group. Data represent two to four independent experiments.

Our results revealed that at baseline, there were no significant differences in the number of these immune cell populations between *CD11c*^*PLXND1 KO*^ and *PLXND1*^*fl/fl*^ mice in the lungs, spleen, lymph nodes, and blood (Fig 1D–1G). These findings indicate that the absence of *PLXND1*, specifically in CD11c+ cells, does not impact immune cell composition in different tissues under steady-state conditions.

### *PLXND1* deficiency in CD11c+ DC exacerbates airway hyperresponsiveness upon HDM exposure

**Fig 2:**
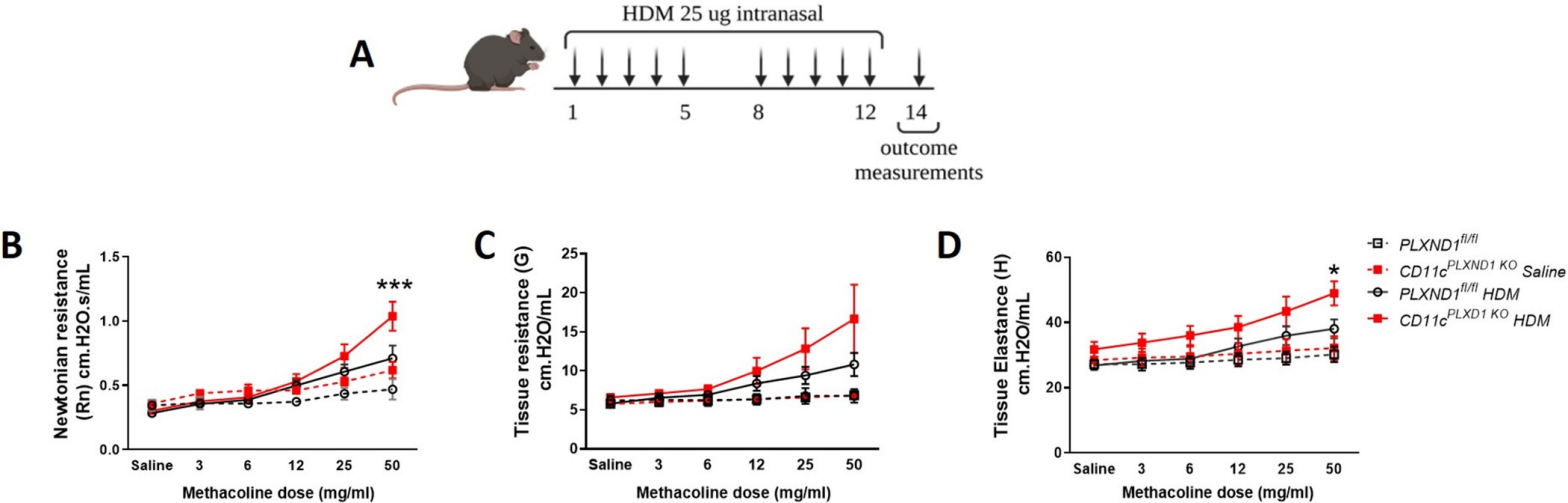
*PLXND1* deletion in CD11c+ DC enhances airway hyperresponsiveness. **(A)** The allergic airway disease model was induced by intranasal exposure to HDM for two weeks; control mice received saline. *CD11c*^*PLXND1 KO*^ and WT mice underwent a tracheotomy followed by a methacholine challenge to measure **(B)** airway resistance, **(C)** tissue resistance, and **(D)** tissue elastance. Results are shown as mean ± SEM, representing three to five mice per group. Data represent two to four independent experiments. *p<0.05, ***p<0.001 by 2-way ANOVA.

*CD11c*^*PLXND1 KO*^ and *PLXND1*^*fl/fl*^ (wild type) mice were exposed to HDM allergen for five consecutive days over two weeks (Fig 2A) [28, 29]. Airway resistance (Rn) significantly increased in *CD11c*^*PLXND1 KO*^ mice compared to *PLXND1*^*fl/fl*^ mice (Fig 2B), while no difference was detected in tissue resistance (G) (Fig 2C). Airway resistance refers to the opposition to airflow in the airways, particularly in the smaller air passages (bronchioles). Increased airway resistance indicates narrowed airways, resulting from inflammation, smooth muscle constriction, and mucus production, leading to breathing difficulties [30].

Tissue elastance (H), which reflects the stiffness or rigidity of the lung tissue, also significantly increased in *CD11c*^*PLXND1 KO*^ mice compared to *PLXND1*^fl/fl^ mice (Fig 2D). Tissue elastance measures the force required to change lung tissue volume, with changes often associated with structural alterations in the airway walls, such as thickening of the smooth muscle layer or collagen deposition, contributing to airway remodeling and decreased lung function (36).

These findings indicate that the absence of *PLXND1* in CD11c+ DCs exacerbates HDM-induced airway hyperresponsiveness in the acute HDM model of allergic asthma.

### *PLXND1* deficiency in CD11c+ DC enhances airway inflammation upon HDM exposure

**Fig 3:**
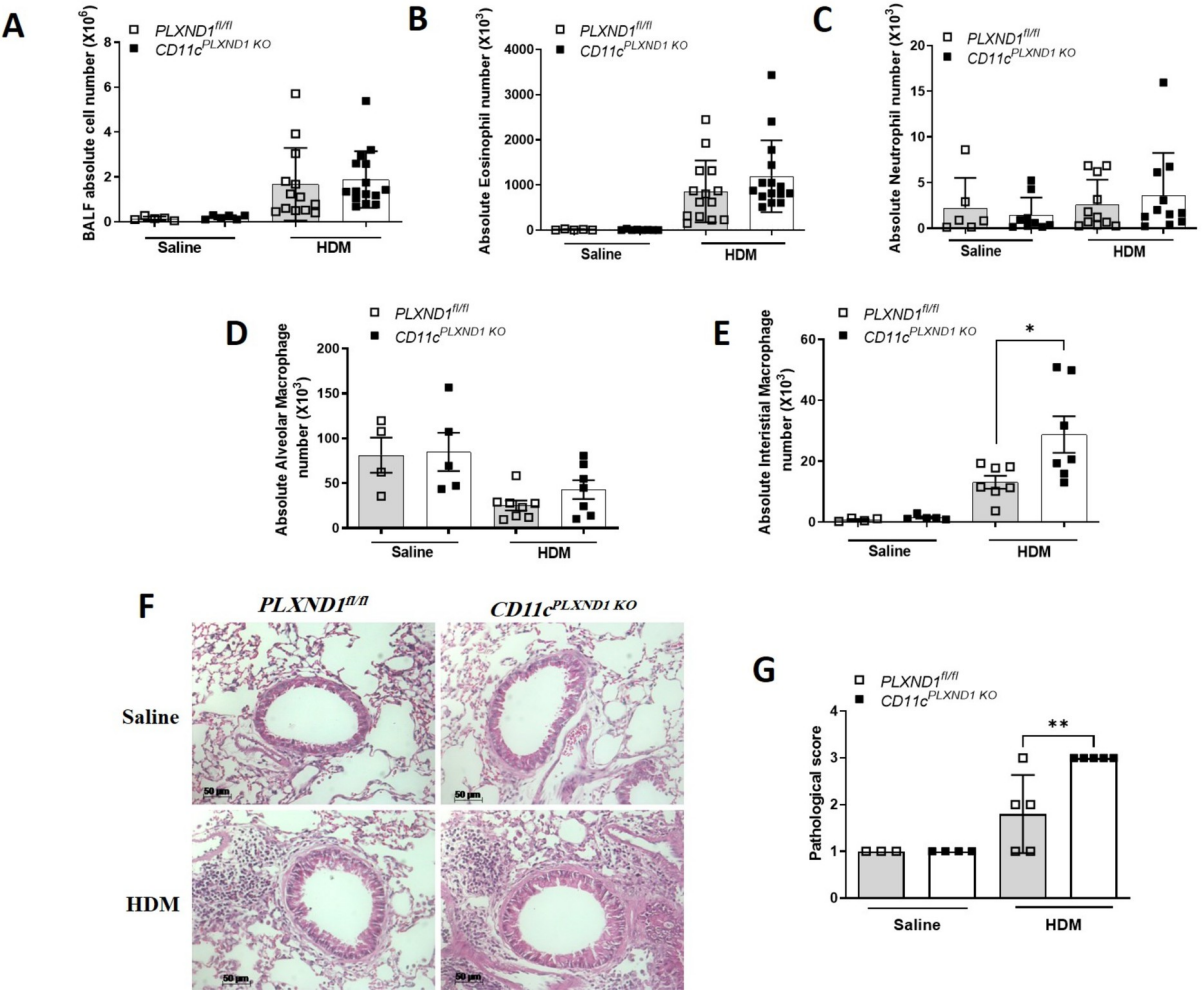
*PLXND1* ablation in CD11c+ DC increases lung inflammation. **(A)** Total BALF cells, **(B)** eosinophils, **(C)** neutrophils, **(D)** alveolar macrophages, and **(E)** interstitial macrophages were quantified using flow cytometry. **(F)** Lung inflammation was evaluated with H&E staining, and **(G)** results were reported as pathological scores. Scale bars: 50um. Data represent mean ± SEM. Representative of three to five mice per group. Data represent two to four independent experiments. *p<0.05, **p<0.01 by 2-way ANOVA.

To assess the impact of *PLXND1* ablation in CD11c+ cells on airway inflammation and cell recruitment into the lungs, we investigated the total number of cells in BALF and the presence of neutrophils, eosinophils, and macrophages using flow cytometry [14]. We found that the numbers of total cells, eosinophils, neutrophils, and alveolar macrophages in the BALF were not significantly altered in HDM-treated *CD11c*^*PLXND1 KO*^ mice compared to *PLXND1*^*fl/fl*^ mice (Fig 3A–3D). However, the number of interstitial macrophages significantly increased in *CD11c*^*PLXND1 KO*^ compared to *PLXND1*^*fl/fl*^ mice (Fig 3E).

In contrast to the BALF, H&E staining of lung tissue sections demonstrated increased recruitment of inflammatory cells in the lungs of *CD11c*^*PLXND1 KO*^ mice compared to *PLXND1*^*fl/fl*^ mice (Fig 3F and 3G). These findings collectively indicate that selective deletion of *PLXND1* in CD11c+ DCs significantly enhances inflammation during asthma, as evidenced by increased recruitment of inflammatory cells and overall histopathological changes in lung tissue.

### Ablation of *PLXND1* in CD11c+ DC affects the production of CCL2/MCP1

**Fig 4:**
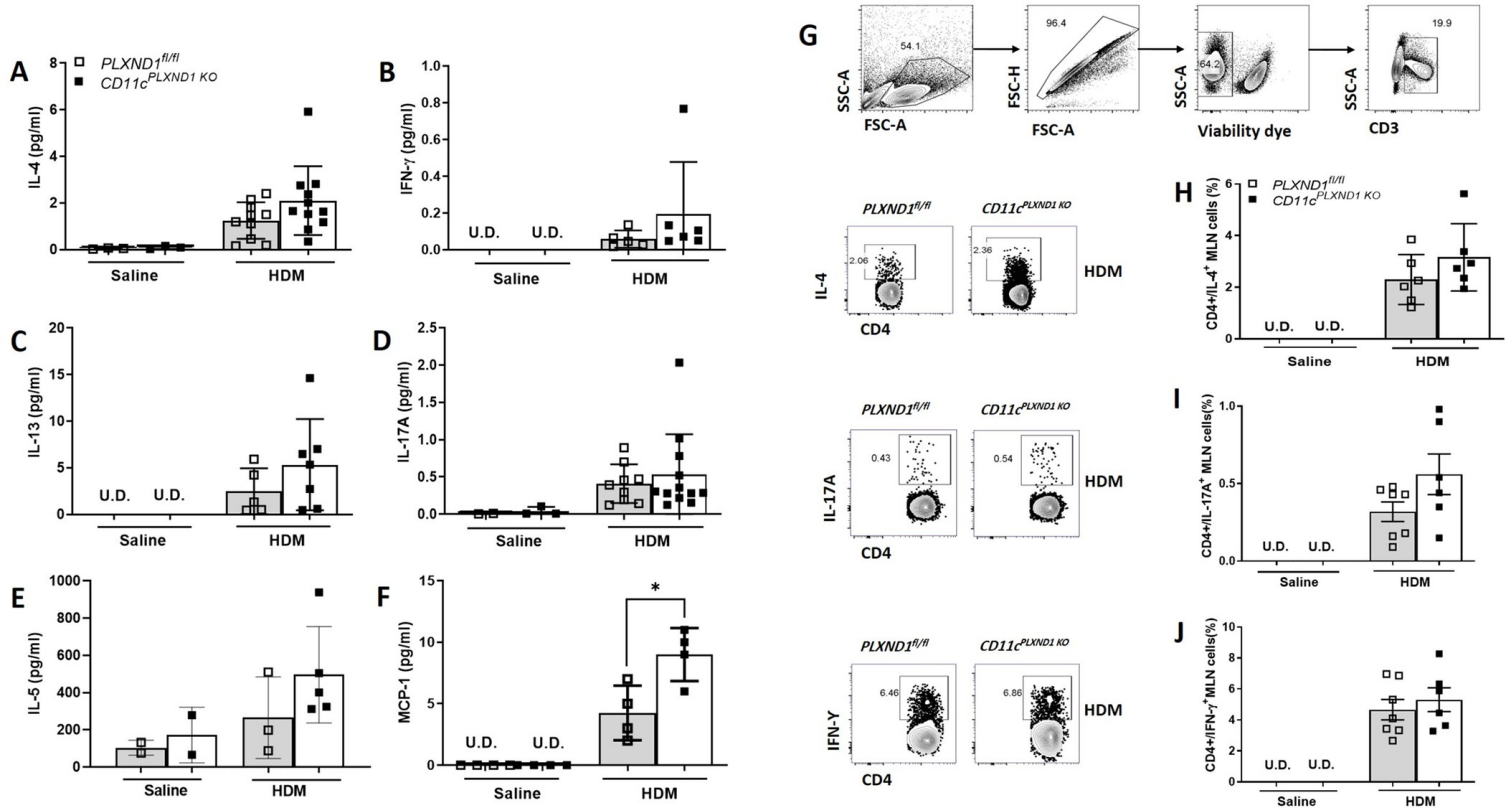
*PLXND1* deficiency in CD11c+ DCs affects CCL2/MCP1 production in allergic asthma. Levels of **(A)** IL-4, **(B)** IFN-γ, **(C)** IL-13, **(D)** IL-17A, **(E)** IL-5, and **(F)** MCP-1 were measured by mesoscale ELISA in BALF supernatants from *CD11c*^*PLXND1 KO*^ and WT mice after HDM exposure. FACS analysis using specific antibodies characterized T cell-derived cytokines from stimulated lymph node cells of *CD11c*^*PLXND1 KO*^ and WT mice. **(G)** The gating strategy included lymphocytes, single cells, viable, and CD3+ T lymphocytes. T cell-derived cytokines were assessed by gating on CD4+ cells and measuring **(H)** IL-4, **(I)** IL-17A, and **(J)** IFN-γ. Data represent mean ± SEM. Representative of three to five mice per group. Data represent two to four independent experiments. *p<0.05 by 2-way ANOVA.

Various cytokines, particularly Th2/Th17 cytokines, play critical roles in the induction and exacerbation of inflammation and remodeling during allergic asthma [31]. We investigated the levels of inflammatory cytokines in the BALF of *CD11c*^*PLXND1 KO*^ and *PLXND1*^*fl/fl*^ mice. Although there was a slight increase in the levels of cytokines in the BALF of *CD11c*^*PLXND1 KO*^, the deletion of *PLXND1* in CD11c+ DC did not statistically affect the production of IL-4, IL-5, IL-13, IL-17A, and IFN-γ in the BALF of *CD11c*^*PLXND1 KO*^ mice compared to *PLXND1*^*fl/fl*^ mice following HDM challenge (Fig 4A–4E). However, the levels of CCL2/MCP-1 (Monocyte chemoattractant protein-1) were significantly higher in *CD11c*^*PLXND1 KO*^ mice compared to *PLXND1*^*fl/fl*^ mice (Fig 4F).

To further assess the T cell subsets and their cytokine production, mediastinal lymph nodes (MLN) were harvested from *CD11c*^*PLXND1 KO*^ and *PLXND1*^*fl/fl*^ mice. MLN cells were stimulated *ex vivo* with a stimulation cocktail containing PMA, ionomycin, and the protein transport inhibitor brefeldin-A and then analyzed using surface and intracellular markers by flow cytometry [20]. IL-4, IL-17A, and IFN-γ levels did not change significantly after stimulation of MLN cells from *CD11c*^*PLXND1 KO*^ compared to WT mice (Fig 4H–4J).

These results suggest that except for CCL2/MCP1, the lack of *PLXND1* in CD11c+ DC does not significantly impact the biosynthesis and release of IL-4, IL-5, IL-13, IL-17A, and IFN-γ in this HDM model of allergic asthma.

### Total and HDM-specific serum IgE levels are enhanced in response to the ablation of *PLXND1* in CD11c+ DC

**Fig 5:**
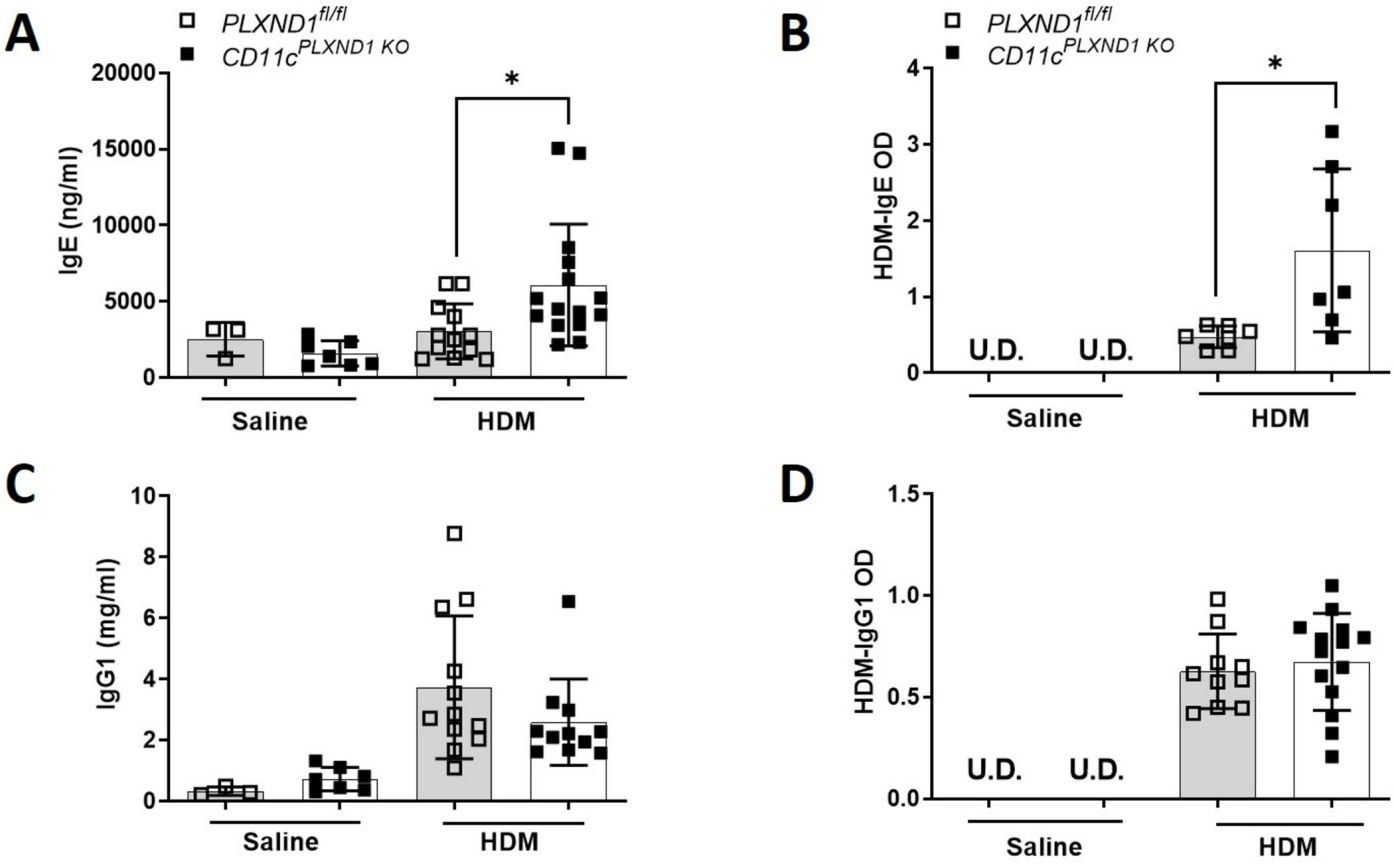
*PLXND1* deficiency in CD11c+ DC increases IgE levels in allergic asthma. After intranasal sensitization and challenge with either saline or HDM, serum samples were collected from *CD11c*^*PLXND1 KO*^ and WT mice. Then, the levels of **(A)** total and **(B)** HDM-specific IgE, as well as **(C)** total and **(D)** HDM-specific IgG1, were measured using ELISA. Data are presented as mean with SEM. All data are representative of three to five mice per group. Data represent two to four independent experiments. 2-Way ANOVA *p<0.05.

Using ELISA, we measured the production of total and HDM-specific Igs in serum isolated from *CD11c*^*PLXND1 KO*^ and *PLXND1*^*fl/fl*^ mice. The total and HDM-specific IgE levels significantly increased in *CD11c*^*PLXND1 KO*^ mice compared to *PLXND1*^*fl/fl*^ mice upon HDM challenge (Fig 5A and 5B). However, there was no difference in total and HDM-specific IgG1 levels following HDM exposure (Fig 5C and 5D). These results suggest that the absence of *PLXND1* on CD11c+ DC enhances IgE levels, which may contribute to the exacerbated allergic reaction in asthma.

### *PLXND1* deletion in CD11c+ DC leads to increased CD11c+ MHCII ^high^ CD11b+ recruitment into the lungs

Lung DCs comprise heterogeneous populations, including conventional DCs categorized into CD11b+ (cDC2) and CD103+ (cDC1) subtypes. CD11b+ cDC2 cells are involved in Th2/Th17 priming and exacerbate atopic responses in the airways, while CD103+ cDC1 cells act as tolerogenic cells and ameliorate inflammation following exposure to HDM [32].

**Fig 6:**
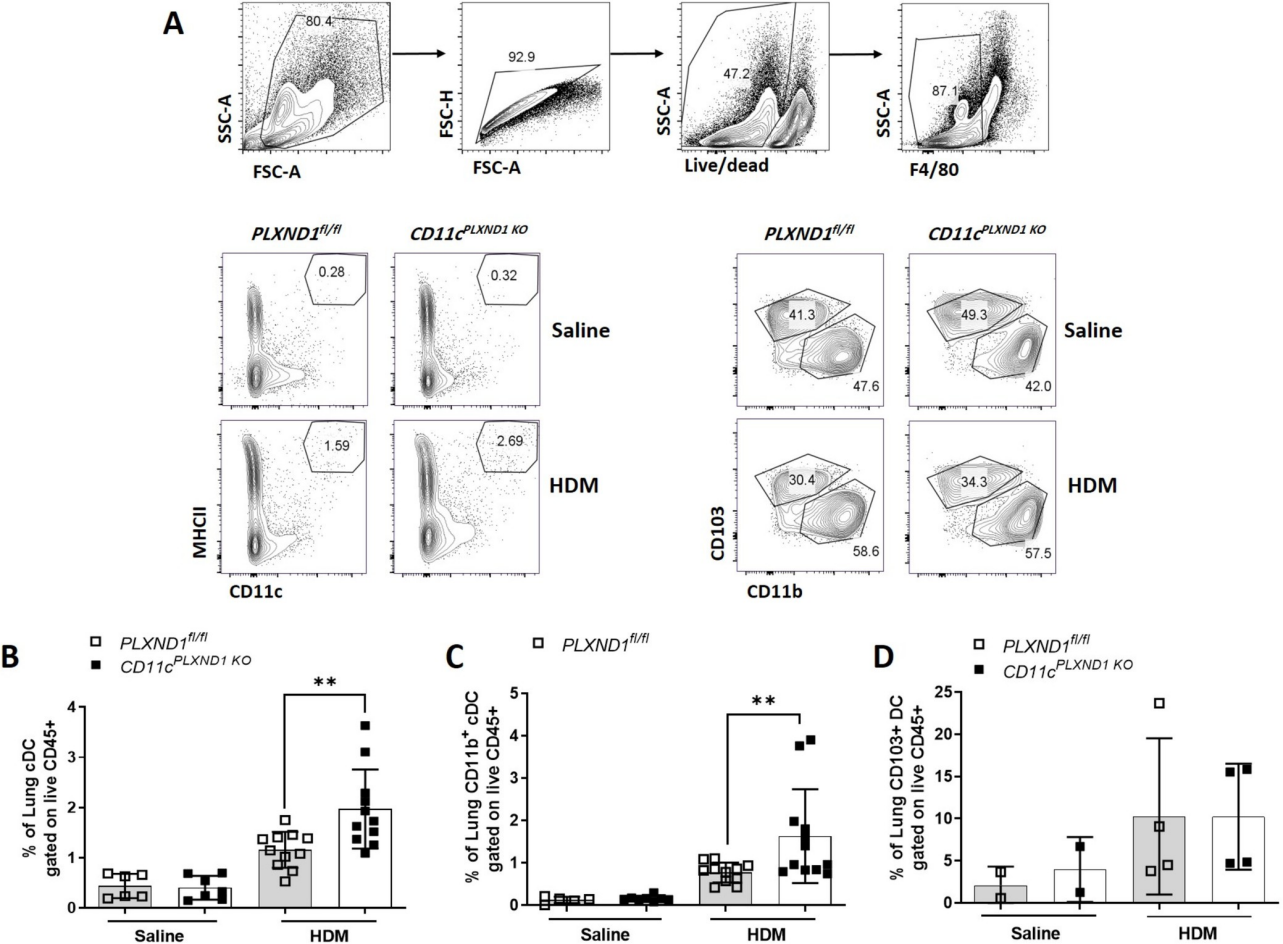
*PLXND1* deletion in CD11c+ DC increases the cDCs recruitment into the lungs. Lungs were harvested from *CD11c*^*PLXND1* KO^ and WT mice after intranasal saline or HDM exposure. **(A)** Pulmonary DC subsets were characterized by flow cytometry, excluding doublets, dead cells, and macrophages (F4/80+). **(B)** Number of pulmonary cDCs (MHCII^high^/CD11c+) and subsets of **(C)** CD11b+ and **(D)** CD103+ DCs were compared between *CD11c*^*PLXND1 KO*^ and *PLXND1*^fl/fl^ mice. Data are presented as mean ± SEM, pre-gated on CD45+. Representative of three to five mice per group. Data represent two to four independent experiments. **p<0.01 by 2-way ANOVA.

We observed a significant increase in the number of cDCs (CD11c+/MHCII+) in the airways of *CD11c*^*PLXND1 KO*^ mice compared with *PLXND1*^*fl/fl*^ mice (Fig 6B). Additionally, the number of CD11c+/MHCH^high^ CD11b+ was significantly higher than CD11c+/MHCH^high^ CD103+ cells following the HDM challenge in *CD11c*^*PLXND1 KO*^ mice (Fig 6C and 6D).

These findings demonstrated that the lack of *PLXND1* in DC increased the recruitment of pulmonary cDCs, mostly CD11b+ DC subtype, which may be linked to the enhanced CCL-2/MCP-1 levels observed in the BALF of *CD11c*^*PLXND1 KO*^ mice.

### Deletion of *PLXND1* in CD11c+ DC enhances IgE levels *ex vivo*

**Fig 7:**
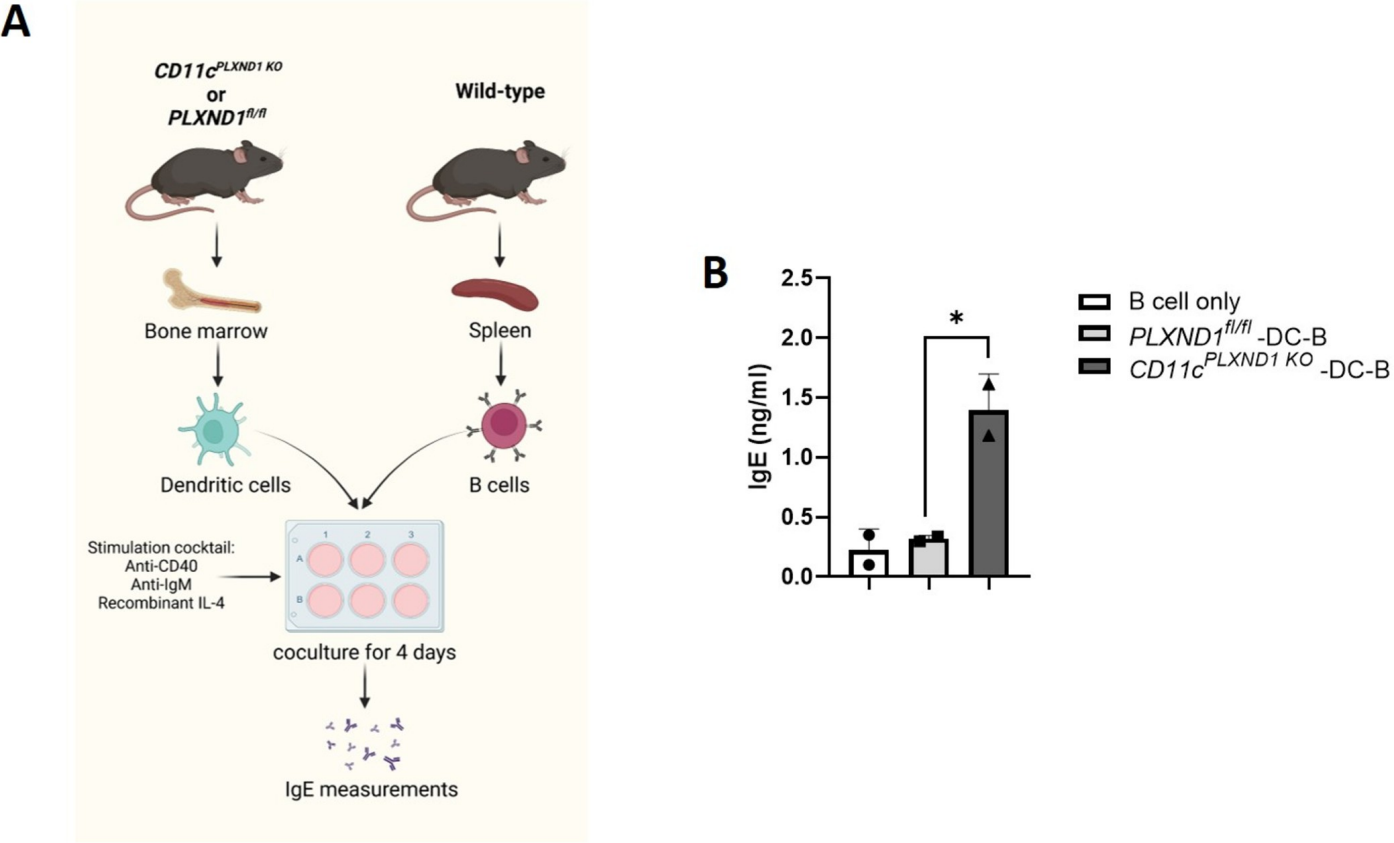
*PLXND1* deficiency in CD11c+ DC induces IgE production *ex vivo*. (**A & B**) Bone marrow-derived DCs from *CD11c*^*PLXND1 KO*^ and *PLXND1*^fl/fl^ mice were co-cultured with splenic B cells isolated from WT mice to assess IgE levels *ex vivo*. Supernatants from DC-B cell co-cultures were collected, and IgE levels were measured using ELISA. Data represent mean ± SEM. Representative of two mice per group. Data represent one experiment. *p<0.05 by 1-way ANOVA.

To further evaluate the impact of *PLXND1* deficiency in CD11c+ DC on B cell function and IgE production, conventional DCs were differentiated from the bone marrow of *CD11c*^*PLXND1 KO*^ and *PLXND1*^*fl/fl*^ mice and co-cultured with isolated splenic B cells from WT mice *ex vivo* [23] (Fig 7A). Notably, IgE levels were significantly higher in the co-cultures with DCs isolated from *CD11c*^*PLXND1 KO*^ mice than those with DCs from *PLXND1*^fl/fl^ mice (Fig 7B).

Considering the crucial role of costimulatory molecules in initiating immune responses and Ig class switching by DCs [33, 34], we also assessed the levels of costimulatory molecules in splenic CD11c+ MHCII^high^ CD103+ and CD11c+ MHCII^high^ CD11b+ cells. No differences in the expression of costimulatory molecules, including CD40, CD80, CD86, BAFF, APRIL, and PDL-1, were observed between *CD11c*^*PLXND1 KO*^ and *PLXND1*^fl/fl^ mice upon HDM challenge (data not shown). This aligns with findings by *Eda K*. *Holl et al*., who showed that the absence of *PLXND1* in DCs did not affect their ability to upregulate costimulatory molecules [35].

These results suggest that the lack of *PLXND1* in DCs enhances IgE production by B cells, which may explain our model’s exacerbated allergic asthma reactions.

### The absence of *PLXND1* in CD11c+ DCs exacerbates goblet cell hyperplasia and increases collagen3 gene expression upon HDM exposure

**Fig 8:**
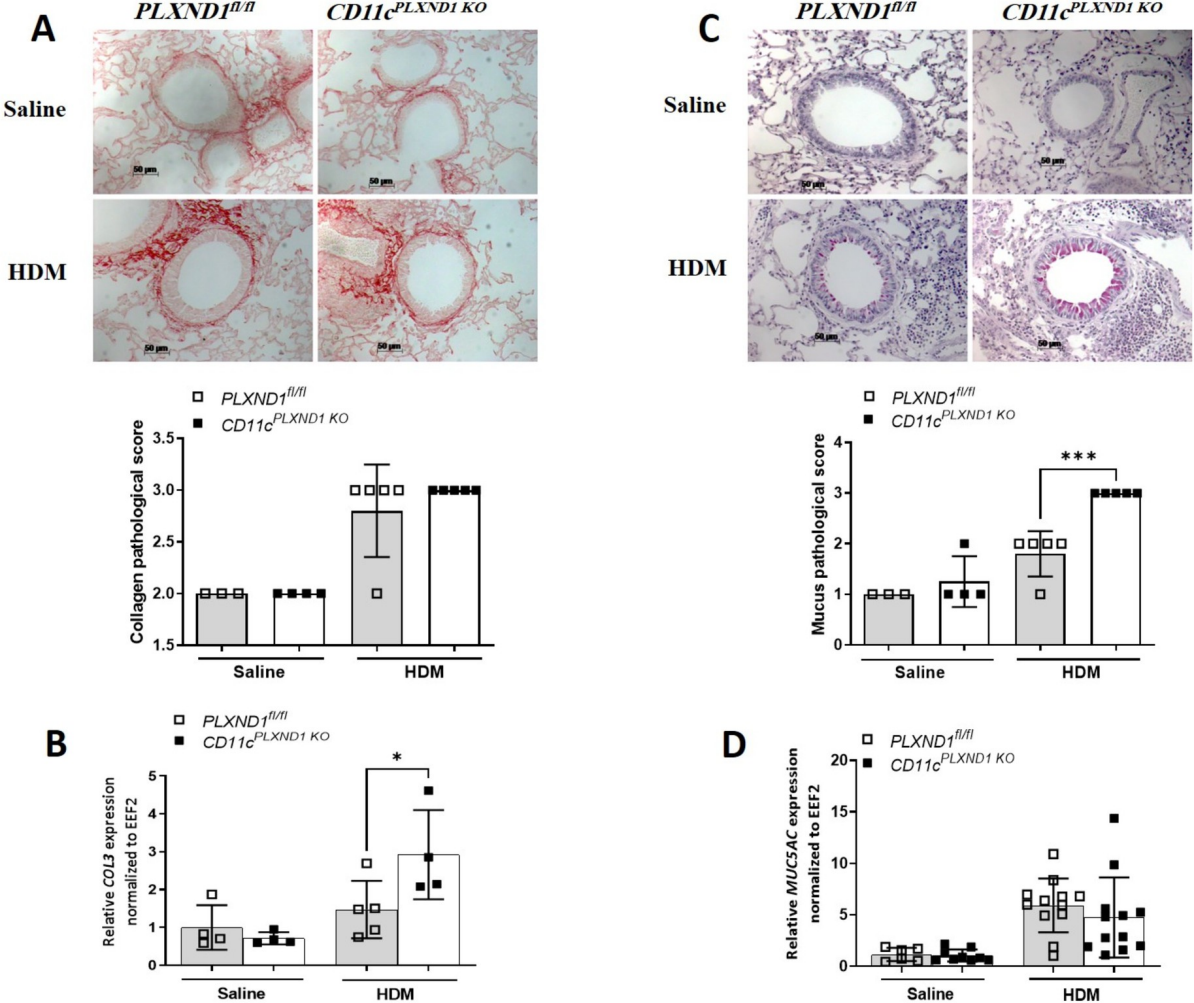
*PLXND1* deletion in CD11c+ DC enhances structural cell changes and airway remodeling. **(A)** Collagen deposition and **(C)** mucus production were visualized using Sirius red and PAS staining. **(B)** Collagen 3 (*COL3*) and **(D)** *MUC5AC* expression were analyzed by real-time PCR in *CD11c*^*PLXND1 KO*^ and *PLXND1*^*fl/fl*^ mice. Scale bars: 50um. Results are shown as mean ± SEM, representing three to five mice per group. Data represent two to four independent experiments. *p<0.05, ***p<0.001 by 2-way ANOVA.

Given that goblet cell proliferation and sub-epithelial fibrosis contribute to airway remodeling in allergic asthma [36], we investigated whether the absence of *PLXIND1* in CD11c+ DC affects mucin production and collagen deposition. Mucus production and collagen deposition were visualized using PAS and Sirius red staining on lung tissue sections. Our results revealed significantly higher mucus production, but not collagen deposition, in *CD11c*^*PLXND1 KO*^ mice compared to *PLXND1*^fl/fl^ mice (Fig 8A and 8C).

To confirm these observations, we assessed the expression of the *COL3* and *Muc5AC* genes in lung tissue. *CD11c*^*PLXND1 KO*^ mice exposed to HDM showed significantly higher *COL3* gene expression than *PLXND1*^fl/fl^ mice (Fig 8B). However, the two groups had no significant difference in *Muc5AC* gene expression (Fig 8D).

These findings indicate that the ablation of *PLXND1* in CD11c+ DCs leads to increased airway remodeling during allergic asthma, evidenced by enhanced mucus production and higher *COL3* gene expression.

## Discussion

In this study, we addressed the impact of *PLXND1* deficiency in CD11c+ DC in an HDM allergic model of asthma [19]. We demonstrated that the lack of *PLXND1* in CD11c+ DC exacerbates airway hyperresponsiveness (AHR) parameters, such as airway resistance and tissue elastance. We also observed higher mucus production and an increase in the collagen gene expression in the lungs of *CD11c*^*PLXND1 KO*^ DC compared to WT counterparts, suggesting the role of CD11c+ DC *PLXND1* deficiency in airway remodelling. Moreover, we found higher airway inflammation, particularly an enhanced number of interstitial macrophages and elevated CCL-2/MCP-1 levels in the BALF of *CD11c*^*PLXND1 KO*^ DC mice. The absence of *PLXND1* in CD11c+ DC resulted in higher total and HDM-specific serum IgE levels and enhanced recruitment of CD11c+ MHCII^high^ CD11b+ to the lungs. Mechanistically, co-culture of B cells with DC from *CD11c*^*PLXND1 KO*^ DC mice led to significantly higher IgE levels *ex vivo* compared to the DC isolated from WT mice. Our data highlighted that the plexinD1 in CD11c+ DC is critical in modulating allergic asthma features, including AHR, remodelling and inflammation.

DCs play a central role in recognizing allergens in the airways and priming B cells, producing IgE antibodies, thereby triggering subsequent allergic responses in individuals with IgE-exacerbated asthma [37]. DCs, by capturing and processing allergens, prime the T cells to recognize and respond to potential stimuli like HDM [38]. Once encountered, DCs migrate to mediastinal lymph nodes, presenting allergens to naive T cells [38]. Crucially, DCs promote the differentiation of naive T cells into Th2 cells, a subset specialized in orchestrating allergic reactions [38]. By releasing specific cytokines, like IL-4 and IL-13, DCs drive the activation and proliferation of Th2 cells, stimulating B cells to differentiate into IgE-producing plasma cells [38]. The resulting IgE antibodies circulate in the bloodstream and tissues, awaiting re-exposure to the same allergen [39]. Upon subsequent encounters, IgE binds to mast cells and basophils, releasing inflammatory mediators like histamine and leukotrienes [39]. This cascade of events leads to airway inflammation, smooth muscle contraction, mucus production, and ultimately, bronchoconstriction, precipitating asthma symptoms [39]. PlexinD1 is highly expressed in lung and bone marrow-derived DCs and mature and immature DCs [17, 35, 40]. Notably, plexinD1 is involved in B cells homing into germinal centers, induction of humoral responses, regulation of long-lived bone marrow plasmacytes, and recall of humoral memory responses [41].

Our findings demonstrate that *PLXND1* ablation in CD11c+ dendritic cells (DCs) leads to increased recruitment of conventional DCs (cDCs) in the lungs, with a notable rise in CD11c+ MHCII^high^ CD11b+ cells compared to CD11c+ MHCII^high^ CD103+ cells. These results align with our previous studies, which reported an elevated presence of CD11b+ DCs relative to CD103+ DCs in the airways of Sema3E knockout (KO) mice, both at baseline and following HDM sensitization (17, 18). This shift in DC subpopulation likely contributes to the exacerbated Th2/Th17 immune response observed in our models (17). The absence of *PLXND1* skews the DC population towards a pro-inflammatory phenotype, contributing to exacerbated asthma features.

CD11b+ DCs are associated with Th2/Th17 immunity, driving IgE production, while CD103+ DCs promote Th1 responses and induce tolerance, inducing IgA production in response to inhaled allergens [42–44]. In this study, we observed elevated total and HDM-specific IgE levels in the serum of *CD11c+ PLXND1 KO* mice compared to wild-type mice. Co-culturing BMDCs from *CD11c+ PLXND1 KO* mice with B cells from wild-type mice resulted in higher IgE *ex vivo* than DCs isolated from wild-type mice. These findings agree with the effect of Sema3E deficiency and *PLXND1* deficiency in Cx3cr1 interstitial macrophages on IgE production [14, 20]. However, the exact mechanism by which plexinD1 in DCs can affect IgE production needs further investigation. Our data suggest that the absence of the *PLXND1* in CD11c+ DC can regulate IgE production by the B cells.

Airway epithelial cells (AECs) have the potential to regulate DCs and macrophages in the lungs by expressing a variety of molecules that can modulate their behavior either positively or negatively through direct and indirect mechanisms [45, 46]. AECs are a significant source of CCL-2/MCP-1 within the lungs, as observed in human asthmatic patients subjected to allergen challenges [47]. CCL-2/MCP-1 plays a pivotal role in the infiltration and migration of monocytes/macrophages to the lungs [47, 48]. In this study, elevated levels of CCL-2/MCP-1 were observed in the BALF of *CD11c*^*PLXND1 KO*^ DC mice, accompanied by an increased number of interstitial macrophages and conventional type-2 DCs compared to their wild-type counterparts.

TNF can stimulate the production of CCL-2/MCP-1 by epithelial cells via the MAPK signalling pathway [49]. DCs and macrophages can produce TNF during the initial stages of immune responses [50, 51]. Furthermore, our prior study has shown that plexinD1 on macrophages modulates TNF production through the MAPK, STAT, and NF-κB signaling pathways [52]. In the current study, it is plausible to hypothesize that plexinD1-deficient CD11c+ DCs induce the production of CCL-2/MCP-1 by airway epithelial cells, subsequently promoting the infiltration of interstitial macrophages into lung tissue in a TNF-dependent manner. Alternatively, allergen exposure itself can induce CCL-2/MCP-1 in epithelial cells. However, additional investigations are necessary to comprehensively understand the mechanisms through which plexinD1 in CD11c+ DCs regulates the production of CCL-2/MCP-1 by airway epithelial cells. In summary, our study underscores the influence of the plexinD1 complex on CD11c+ DCs in shaping airway inflammation during allergic asthma.

Airway hyperresponsiveness (AHR) is the most characteristic clinical feature of asthma, primarily induced by airway inflammation [53]; activated pulmonary DCs trigger the latter through the induction of a Th2/Th17 response [54]. This study demonstrated that the absence of *PLXND1* in CD11c+ DCs exacerbated bronchial hyperreactivity, including airway resistance (Rn) and tissue elastance (H). These findings align with our previous study, where *PLXND1* deletion in Cx3cr1 interstitial macrophages resulted in aggravated airway resistance (Rn) [20]. Furthermore, we previously revealed worsened AHR parameters, such as airway resistance (Rn), tissue resistance (G), and tissue elastance (H), in allergic asthma with a global absence of Sema3E, the canonical ligand for plexinD1 [13–16, 18, 19].

Allergen encounters lead to significant recruitment of DCs into the airways [55]. These DCs activate various subtypes of T cells, leading to enhanced recruitment of immune cells and production of proinflammatory cytokines, such as IL-4, IL-5, IL-9, IL-13, IL-17A, and IFN-γ [54]. These cytokines can trigger smooth muscle cells and induce AHR [31, 54]. Interestingly, specific elimination of conventional DCs can prevent allergic airway inflammation and AHR [56, 57]. In this study, the number of conventional DCs, particularly CD11c+ MHCII^high^ CD11b+, responsible for inducing type 2 inflammation, increased in the lungs in response to *PLXND1* deletion in CD11c+ DC. Although type 2 cytokine levels showed no difference, we observed a significantly higher level of CCL-2/MCP-1 in our CD11c+ DC *PLXND1* KO model, which led us to speculate that the ablation of *PLXND1* in DCs by increasing inflammation, induced AHR in our model. CCL-2/MCP-1 has been shown to induce AHR by directly activating and degranulating mast cells [58, 59]. Notably, anti-MCP-1 antibodies inhibited methacholine-induced AHR and reduced histamine release into the BALF of the cockroach-induced allergic model [58]. Altogether, the regulated recruitment of DC subsets or modulation of their functions by plexinD1 may be linked to the increased AHR observed in our CD11c+ DC *PLXND1* KO model. Further studies are needed to understand how plexinD1 in the CD11c+ DC regulates airway resistance and tissue elastance.

Collagen deposition is considered an essential aspect of asthma [60], and along with the excessive mucin production by goblet cells, it reduces the radius of the airways, restraining airflow and resulting in airway resistance in asthma [61]. We demonstrated that the ablation of *PLXND1* in CD11c+ DC enhanced collagen gene expression, and a significant increase in mucus production in the airways was observed. These findings align with our previous studies, where the deletion of *PLXND1* in Cx3cr1 interstitial macrophages [20] and the global absence of Sema3E resulted in increased collagen deposition in the lamina reticularis, the expression of *Muc5AC* and *Muc5b*, and hypersecretion of mucus in the airways [14, 16, 20].

The exact mechanism of how plexinD1 participates in fibrosis during asthma remains unclear. However, DCs, through interactions with other inflammatory cell types and their mediators, can contribute to airway remodeling [62]. In this study, the number of interstitial macrophages and the levels of CCL-2/MCP-1 significantly increased in response to the deletion of *PLXND1* in CD11c+ DC. CCL-2/MCP-1 stimulates fibroblasts, the cells responsible for producing collagen and other extracellular matrix components, leading to increased production and deposition of collagen [63], thus contributing to the fibrotic remodelling of lung tissue [59]. Furthermore, CCL-2/MCP-1 has been shown to induce the differentiation of blood-recruited fibroblasts into myofibroblasts [64], contributing to tissue scarring and fibrosis. Lastly, CCL-2/MCP-1 can interact with other pro-fibrotic factors, such as transforming growth factor-beta (TGF-β), to promote fibrosis synergistically [63]. TGF-β is a potent inducer of fibrosis, and CCL-2/MCP-1 can potentiate its effects [63]. CCL-2/MCP-1 has been found to interact with other proinflammatory cytokines, such as interleukin-13 (IL-13) [65, 66] and activates p44/42MAPK, a kinase that plays a crucial role in mucin regulation in the bronchial epithelium [67].

Overall, it is plausible to suggest that the impact of CD11c+ DC *PLXND1* KO on airway remodelling and fibrosis could be mediated either by direct interaction of CCL-2/MCP-1 with fibroblasts/myofibroblasts or indirectly by increased recruitment of interstitial macrophages (i.e., M2 macrophages) into the lung, which is associated with pulmonary fibrosis [68]. PlexinD1 signalling in CD11c+ DC is critical for remodelling events during asthma, as evidenced by higher mucus and collagen levels in the airways and increased stiffness or tissue elastance in our model.

Our study highlights the significant role of *PLXND1* in CD11c+ DC in the context of allergic asthma. It is important to note that complete knockout models of *PLXND1* result in embryonic lethality [69], underscoring the complexity of *PLXND1* signaling and suggesting involvement of multiple ligands. In addition to Sema3E, other semaphorins, such as Sema3C and the transmembrane protein Sema4A, have been reported to interact with plexinD1, influencing its signaling pathways [70–74]. Our study did not explore the potential redundancy and compensatory mechanisms involving these ligands, but it could have significant implications.

For instance, Sema4A is known to regulate immune cell functions. It is highly expressed in dendritic cells, suggesting that it could play a crucial role in modulating immune responses in our model [73, 74]. Sema4A significantly enhances T-cell proliferation, cytokine production, and the expression of activation markers (CD25 and CD69), confirming its role as an activator of T-cell-mediated immunity [74]. Concurrently, Sema4A inhibits endothelial cell migration and tube formation *in vitro* and reduces vascularization in vivo, indicating its anti-angiogenic properties. These effects are mediated through the binding of Sema4A to plexinD1 on endothelial cells, leading to downstream signaling that inhibits angiogenesis [74].

Furthermore, a study by *Mogie G et al*. [73] explores the potential of Sema4A as both a therapeutic agent and a target for asthma treatment. The study demonstrates that Sema4A modulates critical aspects of the asthmatic response, including AHR, inflammation, and immune cell activation. Administration of Sema4A reduces AHR, eosinophilic inflammation, and type-2 cytokine levels, including IL-4, IL-5, and IL-13, highlighting its therapeutic potential [73]. Additionally, Sema4A treatment alters DC maturation, reduces their ability to activate T-cells, and decreases the polarization of Th2 cells, thereby reducing allergic inflammation in asthma. The study also identifies signaling pathways involving plexinD1 and other receptors mediating the effects of Sema4A [73].

Future studies should investigate the contributions of these additional plexinD1 ligands to better understand the comprehensive role of plexinD1 signaling in dendritic cells and its impact on airway hyperresponsiveness, IgE production, and mucus secretion in allergic asthma.

## Supporting information

S1 Graphical abstract (TIF)

## Abbreviation

AHR: airway hyperresponsiveness
AM: alveolar macrophage
ASM: airway smooth muscle cell
a-SMA: a-smooth muscle actin
BALF: bronchoalveolar lavage fluid
BM: bone marrow
BMDM: bone marrow-derived macrophage
DC: dendritic cell
GATA3: GATA binding protein 3
HDM: house dust mite
IM: interstitial macrophage
IRF-4: interferon regulatory factor 4
KO: knockout
mLN: mediastinal lymph node
PDL2: programmed death-ligand 2
qRT-PCR: quantitative real-time PCR
RORγt: Retinoic Acid Receptor-Related Orphan Receptor Gamma T
Sema3E: semaphorin3E
Treg: regulatory T cell
WT: wild-type

## References

[1] Global, regional, and national deaths, prevalence, disability-adjusted life years, and years lived with disability for chronic obstructive pulmonary disease and asthma, 1990–2015: a systematic analysis for the Global Burden of Disease Study 2015. Lancet Respir Med. 2017;5(9):691–706. doi: 10.1016/S2213-2600(17)30293-X 28822787 PMC5573769

[2] Hamid Q, Tulic M. Immunobiology of asthma. Annu Rev Physiol. 2009;71:489–507. doi: 10.1146/annurev.physiol.010908.163200 19575684

[3] Chung KF. New treatments for severe treatment-resistant asthma: targeting the right patient. Lancet Respir Med. 2013;1(8):639–52. doi: 10.1016/S2213-2600(13)70128-0 24461667

[4] Kolodkin AL, Matthes DJ, Goodman CS. The semaphorin genes encode a family of transmembrane and secreted growth cone guidance molecules. Cell. 1993;75(7):1389–99. doi: 10.1016/0092-8674(93)90625-z 8269517

[5] Mirakaj V, Rosenberger P. Immunomodulatory Functions of Neuronal Guidance Proteins. Trends Immunol. 2017;38(6):444–56. doi: 10.1016/j.it.2017.03.007 28438491

[6] Alto LT, Terman JR. Semaphorins and their Signaling Mechanisms. Methods Mol Biol. 2017;1493:1–25. doi: 10.1007/978-1-4939-6448-2_1 27787839 PMC5538787

[7] Choi YI, Duke-Cohan JS, Ahmed WB, Handley MA, Mann F, Epstein JA, et al. PlexinD1 glycoprotein controls migration of positively selected thymocytes into the medulla. Immunity. 2008;29(6):888–98. doi: 10.1016/j.immuni.2008.10.008 19027330 PMC2615553

[8] Movassagh H, Khadem F, Gounni AS. Semaphorins and Their Roles in Airway Biology: Potential as Therapeutic Targets. Am J Respir Cell Mol Biol. 2018;58(1):21–7. doi: 10.1165/rcmb.2017-0171TR 28817310

[9] Matloubi M, Koussih L, Shan L, Lukawy C, Gounni AS. Targeting the Semaphorin3E-plexinD1 complex in allergic asthma. Pharmacol Ther. 2023;242:108351. doi: 10.1016/j.pharmthera.2023.108351 36706796

[10] Meyer LA, Fritz J, Pierdant-Mancera M, Bagnard D. Current drug design to target the Semaphorin/Neuropilin/Plexin complexes. Cell Adh Migr. 2016;10(6):700–8. doi: 10.1080/19336918.2016.1261785 27906605 PMC5160035

[11] Gu C, Yoshida Y, Livet J, Reimert DV, Mann F, Merte J, et al. Semaphorin 3E and plexin-D1 control vascular pattern independently of neuropilins. Science. 2005;307(5707):265–8. doi: 10.1126/science.1105416 15550623

[12] Worzfeld T, Offermanns S. Semaphorins and plexins as therapeutic targets. Nat Rev Drug Discov. 2014;13(8):603–21. doi: 10.1038/nrd4337 25082288

[13] Movassagh H, Saati A, Nandagopal S, Mohammed A, Tatari N, Shan L, et al. Chemorepellent Semaphorin 3E Negatively Regulates Neutrophil Migration In Vitro and In Vivo. J Immunol. 2017;198(3):1023–33. doi: 10.4049/jimmunol.1601093 27913633

[14] Movassagh H, Shan L, Duke-Cohan JS, Chakir J, Halayko AJ, Koussih L, et al. Downregulation of semaphorin 3E promotes hallmarks of experimental chronic allergic asthma. Oncotarget. 2017;8(58):98953–63. doi: 10.18632/oncotarget.22144 29228740 PMC5716780

[15] Movassagh H, Shan L, Halayko AJ, Roth M, Tamm M, Chakir J, et al. Neuronal chemorepellent Semaphorin 3E inhibits human airway smooth muscle cell proliferation and migration. The Journal of allergy and clinical immunology. 2014;133(2):560–7. doi: 10.1016/j.jaci.2013.06.011 23932461

[16] Movassagh H, Shan L, Duke-Cohan JS, Halayko AJ, Uzonna JE, Gounni AS. Semaphorin 3E Alleviates Hallmarks of House Dust Mite-Induced Allergic Airway Disease. The American journal of pathology. 2017;187(7):1566–76. doi: 10.1016/j.ajpath.2017.03.008 28634005

[17] Movassagh H, Shan L, Koussih L, Alamri A, Ariaee N, Kung SKP, et al. Semaphorin 3E deficiency dysregulates dendritic cell functions: In vitro and in vivo evidence. PLoS One. 2021;16(6):e0252868. doi: 10.1371/journal.pone.0252868 34185781 PMC8241044

[18] Movassagh H, Shan L, Mohammed A, Halayko AJ, Gounni AS. Semaphorin 3E Deficiency Exacerbates Airway Inflammation, Hyperresponsiveness, and Remodeling in a Mouse Model of Allergic Asthma. J Immunol. 2017;198(5):1805–14. doi: 10.4049/jimmunol.1601514 28108561

[19] Tatari N, Movassagh H, Shan L, Koussih L, Gounni AS. Semaphorin 3E Inhibits House Dust Mite-Induced Angiogenesis in a Mouse Model of Allergic Asthma. The American journal of pathology. 2019;189(4):762–72. doi: 10.1016/j.ajpath.2019.01.008 30711489

[20] Aktar A, Shan L, Koussih L, Almiski MS, Basu S, Halayko A, et al. PlexinD1 Deficiency in Lung Interstitial Macrophages Exacerbates House Dust Mite-Induced Allergic Asthma. J Immunol. 2022;208(5):1272–9. doi: 10.4049/jimmunol.2100089 35110420

[21] Merad M, Sathe P, Helft J, Miller J, Mortha A. The dendritic cell lineage: ontogeny and function of dendritic cells and their subsets in the steady state and the inflamed setting. Annu Rev Immunol. 2013;31:563–604. doi: 10.1146/annurev-immunol-020711-074950 23516985 PMC3853342

[22] Choi YI, Duke-Cohan JS, Chen W, Liu B, Rossy J, Tabarin T, et al. Dynamic control of β1 integrin adhesion by the plexinD1-sema3E axis. Proc Natl Acad Sci U S A. 2014;111(1):379–84. 24344262 10.1073/pnas.1314209111 PMC3890783

[23] Obayashi K, Doi T, Koyasu S. Dendritic cells suppress IgE production in B cells. Int Immunol. 2007;19(2):217–26. doi: 10.1093/intimm/dxl138 17208926

[24] Dubois B, Vanbervliet B, Fayette J, Massacrier C, Van Kooten C, Brière F, et al. Dendritic cells enhance growth and differentiation of CD40-activated B lymphocytes. J Exp Med. 1997;185(5):941–51. doi: 10.1084/jem.185.5.941 9120400 PMC2196162

[25] Gounni AS, Spanel-Borowski K, Palacios M, Heusser C, Moncada S, Lobos E. Pulmonary inflammation induced by a recombinant Brugia malayi gamma-glutamyl transpeptidase homolog: involvement of humoral autoimmune responses. Mol Med. 2001;7(5):344–54. 11474580 PMC1950044

[26] Ashcroft T, Simpson JM, Timbrell V. Simple method of estimating severity of pulmonary fibrosis on a numerical scale. J Clin Pathol. 1988;41(4):467–70. doi: 10.1136/jcp.41.4.467 3366935 PMC1141479

[27] Eissa N, Hussein H, Wang H, Rabbi MF, Bernstein CN, Ghia JE. Stability of Reference Genes for Messenger RNA Quantification by Real-Time PCR in Mouse Dextran Sodium Sulfate Experimental Colitis. PLoS One. 2016;11(5):e0156289. doi: 10.1371/journal.pone.0156289 27244258 PMC4886971

[28] Johnson JR, Wiley RE, Fattouh R, Swirski FK, Gajewska BU, Coyle AJ, et al. Continuous exposure to house dust mite elicits chronic airway inflammation and structural remodeling. Am J Respir Crit Care Med. 2004;169(3):378–85. doi: 10.1164/rccm.200308-1094OC 14597485

[29] Fattouh R, Al-Garawi A, Fattouh M, Arias K, Walker TD, Goncharova S, et al. Eosinophils are dispensable for allergic remodeling and immunity in a model of house dust mite-induced airway disease. Am J Respir Crit Care Med. 2011;183(2):179–88. doi: 10.1164/rccm.200905-0736OC 20732990

[30] Lundblad LK. Issues determining direct airways hyperresponsiveness in mice. Front Physiol. 2012;3:408. doi: 10.3389/fphys.2012.00408 23097643 PMC3477826

[31] Lambrecht BN, Hammad H, Fahy JV. The Cytokines of Asthma. Immunity. 2019;50(4):975–91. doi: 10.1016/j.immuni.2019.03.018 30995510

[32] Guilliams M, Ginhoux F, Jakubzick C, Naik SH, Onai N, Schraml BU, et al. Dendritic cells, monocytes and macrophages: a unified nomenclature based on ontogeny. Nat Rev Immunol. 2014;14(8):571–8. doi: 10.1038/nri3712 25033907 PMC4638219

[33] Naito T, Suda T, Suzuki K, Nakamura Y, Inui N, Sato J, et al. Lung dendritic cells have a potent capability to induce production of immunoglobulin A. Am J Respir Cell Mol Biol. 2008;38(2):161–7. doi: 10.1165/rcmb.2007-0237OC 17709597

[34] Gloudemans AK, Lambrecht BN, Smits HH. Potential of immunoglobulin A to prevent allergic asthma. Clin Dev Immunol. 2013;2013:542091. doi: 10.1155/2013/542091 23690823 PMC3649226

[35] Holl EK, Roney KE, Allen IC, Steinbach E, Arthur JC, Buntzman A, et al. Plexin-B2 and Plexin-D1 in dendritic cells: expression and IL-12/IL-23p40 production. PLoS One. 2012;7(8):e43333. doi: 10.1371/journal.pone.0043333 22916243 PMC3419716

[36] Bergeron C, Tulic MK, Hamid Q. Airway remodelling in asthma: from benchside to clinical practice. Can Respir J. 2010;17(4):e85–93. doi: 10.1155/2010/318029 20808979 PMC2933777

[37] Foster B, Metcalfe DD, Prussin C. Human dendritic cell 1 and dendritic cell 2 subsets express FcepsilonRI: correlation with serum IgE and allergic asthma. The Journal of allergy and clinical immunology. 2003;112(6):1132–8. doi: 10.1016/j.jaci.2003.09.011 14657872

[38] Akdis M, Akdis CA. Mechanisms of allergen-specific immunotherapy. The Journal of allergy and clinical immunology. 2007;119(4):780–91. doi: 10.1016/j.jaci.2007.01.022 17321578

[39] Gould HJ, Sutton BJ. IgE in allergy and asthma today. Nat Rev Immunol. 2008;8(3):205–17. doi: 10.1038/nri2273 18301424

[40] Alamri A, Rahman R, Zhang M, Alamri A, Gounni AS, Kung SKP. Semaphorin-3E Produced by Immature Dendritic Cells Regulates Activated Natural Killer Cells Migration. Front Immunol. 2018;9:1005. doi: 10.3389/fimmu.2018.01005 29867980 PMC5954025

[41] Holl EK, O’Connor BP, Holl TM, Roney KE, Zimmermann AG, Jha S, et al. Plexin-D1 is a novel regulator of germinal centers and humoral immune responses. J Immunol. 2011;186(10):5603–11. doi: 10.4049/jimmunol.1003464 21464091 PMC3771081

[42] Plantinga M, Guilliams M, Vanheerswynghels M, Deswarte K, Branco-Madeira F, Toussaint W, et al. Conventional and monocyte-derived CD11b(+) dendritic cells initiate and maintain T helper 2 cell-mediated immunity to house dust mite allergen. Immunity. 2013;38(2):322–35. doi: 10.1016/j.immuni.2012.10.016 23352232

[43] Furuhashi K, Suda T, Hasegawa H, Suzuki Y, Hashimoto D, Enomoto N, et al. Mouse lung CD103+ and CD11bhigh dendritic cells preferentially induce distinct CD4+ T-cell responses. Am J Respir Cell Mol Biol. 2012;46(2):165–72. doi: 10.1165/rcmb.2011-0070OC 21908266

[44] Khare A, Krishnamoorthy N, Oriss TB, Fei M, Ray P, Ray A. Cutting edge: inhaled antigen upregulates retinaldehyde dehydrogenase in lung CD103+ but not plasmacytoid dendritic cells to induce Foxp3 de novo in CD4+ T cells and promote airway tolerance. J Immunol. 2013;191(1):25–9. doi: 10.4049/jimmunol.1300193 23733880 PMC3694746

[45] Lambrecht BN, Hammad H. The airway epithelium in asthma. Nat Med. 2012;18(5):684–92. doi: 10.1038/nm.2737 22561832

[46] Hussell T, Bell TJ. Alveolar macrophages: plasticity in a tissue-specific context. Nat Rev Immunol. 2014;14(2):81–93. doi: 10.1038/nri3600 24445666

[47] Lee YG, Jeong JJ, Nyenhuis S, Berdyshev E, Chung S, Ranjan R, et al. Recruited alveolar macrophages, in response to airway epithelial-derived monocyte chemoattractant protein 1/CCl2, regulate airway inflammation and remodeling in allergic asthma. Am J Respir Cell Mol Biol. 2015;52(6):772–84. doi: 10.1165/rcmb.2014-0255OC 25360868 PMC4491131

[48] Deshmane SL, Kremlev S, Amini S, Sawaya BE. Monocyte chemoattractant protein-1 (MCP-1): an overview. J Interferon Cytokine Res. 2009;29(6):313–26. doi: 10.1089/jir.2008.0027 19441883 PMC2755091

[49] Ho AW, Wong CK, Lam CW. Tumor necrosis factor-alpha up-regulates the expression of CCL2 and adhesion molecules of human proximal tubular epithelial cells through MAPK signaling pathways. Immunobiology. 2008;213(7):533–44. doi: 10.1016/j.imbio.2008.01.003 18656701

[50] Parameswaran N, Patial S. Tumor necrosis factor-α signaling in macrophages. Crit Rev Eukaryot Gene Expr. 2010;20(2):87–103. 21133840 10.1615/critreveukargeneexpr.v20.i2.10 PMC3066460

[51] Trevejo JM, Marino MW, Philpott N, Josien R, Richards EC, Elkon KB, et al. TNF-alpha -dependent maturation of local dendritic cells is critical for activating the adaptive immune response to virus infection. Proc Natl Acad Sci U S A. 2001;98(21):12162–7. doi: 10.1073/pnas.211423598 11593031 PMC59785

[52] Mohammed A, Okwor I, Shan L, Onyilagha C, Uzonna JE, Gounni AS. Semaphorin 3E Regulates the Response of Macrophages to Lipopolysaccharide-Induced Systemic Inflammation. J Immunol. 2020;204(1):128–36. doi: 10.4049/jimmunol.1801514 31776203

[53] Lommatzsch M. Airway hyperresponsiveness: new insights into the pathogenesis. Semin Respir Crit Care Med. 2012;33(6):579–87. doi: 10.1055/s-0032-1325617 23047309

[54] Lambrecht BN, Hammad H. Biology of lung dendritic cells at the origin of asthma. Immunity. 2009;31(3):412–24. doi: 10.1016/j.immuni.2009.08.008 19766084

[55] Bratke K, Lommatzsch M, Julius P, Kuepper M, Kleine HD, Luttmann W, et al. Dendritic cell subsets in human bronchoalveolar lavage fluid after segmental allergen challenge. Thorax. 2007;62(2):168–75. doi: 10.1136/thx.2006.067793 16928719 PMC2111237

[56] Lambrecht BN, Hammad H. The immunology of asthma. Nat Immunol. 2015;16(1):45–56. doi: 10.1038/ni.3049 25521684

[57] Kopf M, Schneider C, Nobs SP. The development and function of lung-resident macrophages and dendritic cells. Nat Immunol. 2015;16(1):36–44. doi: 10.1038/ni.3052 25521683

[58] Campbell EM, Charo IF, Kunkel SL, Strieter RM, Boring L, Gosling J, et al. Monocyte chemoattractant protein-1 mediates cockroach allergen-induced bronchial hyperreactivity in normal but not CCR2-/- mice: the role of mast cells. J Immunol. 1999;163(4):2160–7. 10438957

[59] Blease K, Lukacs NW, Hogaboam CM, Kunkel SL. Chemokines and their role in airway hyper-reactivity. Respir Res. 2000;1(1):54–61. doi: 10.1186/rr13 11667966 PMC59544

[60] Trejo Bittar HE, Yousem SA, Wenzel SE. Pathobiology of severe asthma. Annu Rev Pathol. 2015;10:511–45. doi: 10.1146/annurev-pathol-012414-040343 25423350

[61] Rogers DF. Airway mucus hypersecretion in asthma: an undervalued pathology? Curr Opin Pharmacol. 2004;4(3):241–50. doi: 10.1016/j.coph.2004.01.011 15140415

[62] Lambrecht BN, Hammad H. Taking our breath away: dendritic cells in the pathogenesis of asthma. Nat Rev Immunol. 2003;3(12):994–1003. doi: 10.1038/nri1249 14647481

[63] Gharaee-Kermani M, Denholm EM, Phan SH. Costimulation of fibroblast collagen and transforming growth factor beta1 gene expression by monocyte chemoattractant protein-1 via specific receptors. J Biol Chem. 1996;271(30):17779–84. doi: 10.1074/jbc.271.30.17779 8663511

[64] Quan TE, Cowper S, Wu SP, Bockenstedt LK, Bucala R. Circulating fibrocytes: collagen-secreting cells of the peripheral blood. Int J Biochem Cell Biol. 2004;36(4):598–606. doi: 10.1016/j.biocel.2003.10.005 15010326

[65] Murray LA, Argentieri RL, Farrell FX, Bracht M, Sheng H, Whitaker B, et al. Hyper-responsiveness of IPF/UIP fibroblasts: interplay between TGFbeta1, IL-13 and CCL2. Int J Biochem Cell Biol. 2008;40(10):2174–82. doi: 10.1016/j.biocel.2008.02.016 18395486

[66] Zhu Z, Ma B, Zheng T, Homer RJ, Lee CG, Charo IF, et al. IL-13-induced chemokine responses in the lung: role of CCR2 in the pathogenesis of IL-13-induced inflammation and remodeling. J Immunol. 2002;168(6):2953–62. doi: 10.4049/jimmunol.168.6.2953 11884467

[67] Monzon ME, Forteza RM, Casalino-Matsuda SM. MCP-1/CCR2B-dependent loop upregulates MUC5AC and MUC5B in human airway epithelium. Am J Physiol Lung Cell Mol Physiol. 2011;300(2):L204–15. doi: 10.1152/ajplung.00292.2010 21097527 PMC3043814

[68] Cheng P, Li S, Chen H. Macrophages in Lung Injury, Repair, and Fibrosis. Cells. 2021;10(2). doi: 10.3390/cells10020436 33670759 PMC7923175

[69] Zhang Y, Singh MK, Degenhardt KR, Lu MM, Bennett J, Yoshida Y, et al. Tie2Cre-mediated inactivation of plexinD1 results in congenital heart, vascular and skeletal defects. Dev Biol. 2009;325(1):82–93. doi: 10.1016/j.ydbio.2008.09.031 18992737 PMC2650856

[70] Toyofuku T, Yoshida J, Sugimoto T, Yamamoto M, Makino N, Takamatsu H, et al. Repulsive and attractive semaphorins cooperate to direct the navigation of cardiac neural crest cells. Dev Biol. 2008;321(1):251–62. doi: 10.1016/j.ydbio.2008.06.028 18625214

[71] Yang WJ, Hu J, Uemura A, Tetzlaff F, Augustin HG, Fischer A. Semaphorin-3C signals through Neuropilin-1 and PlexinD1 receptors to inhibit pathological angiogenesis. EMBO Mol Med. 2015;7(10):1267–84. doi: 10.15252/emmm.201404922 26194913 PMC4604683

[72] Smolkin T, Nir-Zvi I, Duvshani N, Mumblat Y, Kessler O, Neufeld G. Complexes of plexin-A4 and plexin-D1 convey semaphorin-3C signals to induce cytoskeletal collapse in the absence of neuropilins. J Cell Sci. 2018;131(9). doi: 10.1242/jcs.208298 29661844

[73] Mogie G, Shanks K, Nkyimbeng-Takwi EH, Smith E, Davila E, Lipsky MM, et al. Neuroimmune semaphorin 4A as a drug and drug target for asthma. Int Immunopharmacol. 2013;17(3):568–75. doi: 10.1016/j.intimp.2013.08.005 23994348 PMC3818409

[74] Toyofuku T, Yabuki M, Kamei J, Kamei M, Makino N, Kumanogoh A, et al. Semaphorin-4A, an activator for T-cell-mediated immunity, suppresses angiogenesis via Plexin-D1. Embo j. 2007;26(5):1373–84. doi: 10.1038/sj.emboj.7601589 17318185 PMC1817636

