# Supplementary figures and images for: CD11c+ dendritic cells PlexinD1 deficiency exacerbates airway hyperresponsiveness, IgE and mucus production in a mouse model of allergic asthma
Source: PLoS One. 2024 Aug 30;19(8):e0309868. doi: 10.1371/journal.pone.0309868 (PMC11364237; doi:10.1371/journal.pone.0309868)

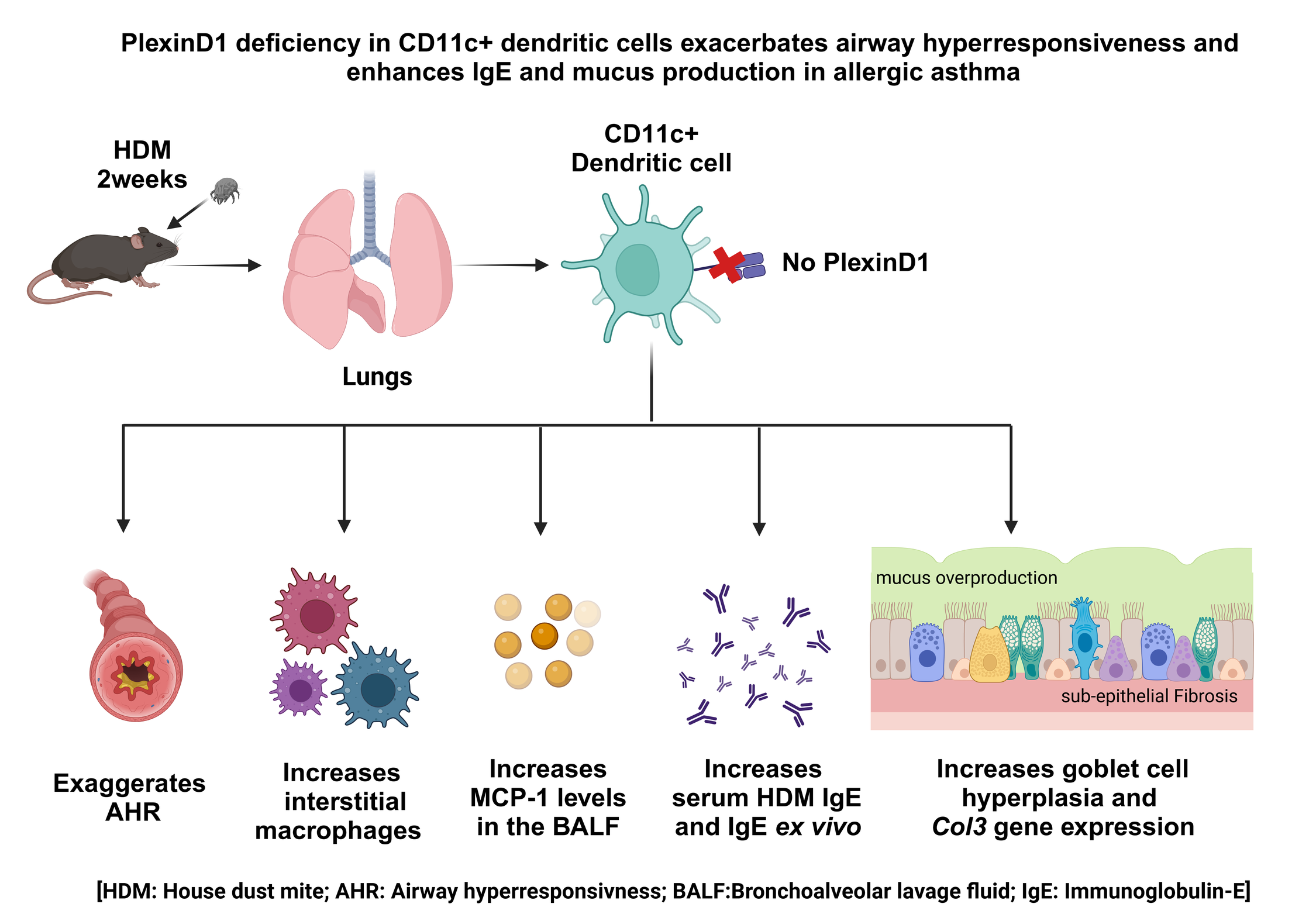

Supplement: S1 Graphical abstract — (TIF) [file pone.0309868.s001.tif]
